# Upregulation of the long non-coding RNA PVT1 promotes esophageal squamous cell carcinoma progression by acting as a molecular sponge of miR-203 and LASP1

**DOI:** 10.18632/oncotarget.15878

**Published:** 2017-03-03

**Authors:** Pin-Dong Li, Jian-Li Hu, Charlie Ma, Hong Ma, Jing Yao, Li-Li Chen, Jing Chen, Tian-Tian Cheng, Kun-Yu Yang, Gang Wu, Wen-Jie Zhang, Ru-Bo Cao

**Affiliations:** ^1^ Cancer Center, Union Hospital, Tongji Medical College, Huazhong University of Science and Technology, Wuhan 430022, China; ^2^ Department of Radiation Oncology, Fox Chase Cancer Center, Philadelphia, PA 19111, USA; ^3^ Cancer Center of Guangzhou Medical University, Guangzhou Medical University, Guangzhou, Guangdong 510059, China; ^4^ Department of Pathology, Shihezi University School of Medicine, Shihezi, Xinjiang 832002, China

**Keywords:** long non-coding RNA, PVT1, LASP1, miR-203, esophageal squamous cell carcinoma

## Abstract

Long non-coding RNAs are a group of non-coding RNAs longer than 200 nucleotides and possess diverse functions and exhibit exquisite cell-specific and developmental dynamic expression patterns. The role of the long non-coding RNA PVT1 in esophageal squamous cell carcinoma remains unsolved. Here, we showed that PVT1 expression is significantly up-regulated in ESCC tumor samples compared with their normal counterparts. Knockdown of PVT1 suppressed tumor growth *in vitro* and *in vivo*. Further studies revealed that silence of PVT1 lead to up-regulation of miR-203, and vice versa. Moreover, LASP1 was found to be downregulated after knockdown of PVT1 and overexpression of LASP1 attenuated the tumor-suppressive roles of PVT1 knockdown. Our results suggest that PVT1 promote ESCC progression via functioning as a molecular sponge for miR-203 and LASP1 and provide the first evidence of dysregulated PVT1/miR-203/LASP1 axis in ESCC.

## INTRODUCTION

Esophageal cancer is one of the most malignant diseases and the sixth most common cause of cancer-related death [1]. Esophageal cancer comprises two common histological subtypes: esophageal adenocarcinoma (EAC) and esophageal squamous cell carcinoma (ESCC)[2]. In Asia, 90% of patients suffered from esophageal cancer was diagnosed as ESCC and China is among the highest risk areas [2, 3]. Despite recent advancements in ESCC treatment including surgery, radiation, and/or chemotherapy, the prognosis is still unsatisfied, and the overall 5-year survival rate is less than 20% [4]. It is, therefore, urgent to uncover the underlying molecular mechanisms and screen useful biomarkers of ESCC.

The combination of various genome-wide approaches has provided evidence that much of the genome serves as regulatory units although with non-protein coding potential [5, 6]. Among which are a new group of non-coding RNA, named as long non-coding RNA (lncRNA) longer than 200 nucleotides. LncRNAs possess diverse functions and exhibiting exquisite cell-specific and developmental dynamic expression patterns [7]. Dysregulation of lncRNAs has been found in various types of carcinomas and serves as tissue-specific oncogenes or tumor suppressors [7–10]. One such lncRNA is plasmacytoma variant translocation 1 (PVT1), which is encoded by the PVT1 gene and localizes downstream of the MYC gene [11]. Recent studies indicated that PVT1 RNA and MYC protein expression correlated in primary human tumours, and copy number of PVT1 was co-increased in more than 98% of MYC copy number-increased cancers [12]. Amplification of PVT1 contributes to the aggressive pathophysiology of ovarian and breast cancer and overexpression of PVT1 is a powerful predictor of tumor progression and overall survival in patients with diverse types of cancer, including gastric cancer [8] and colorectal cancer [12]. However, expression and biological functions of PVT1 in ESCC and the underlying mechanisms remain unsolved.

The Lim and SH3 domain protein (LASP1) is an actin-binding protein [13] and was initially identified from metastatic axillary lymph nodes of patients with breast cancer [14]. LASP1 was reported to be associated with dynamic actin assembly, such as focal contacts, focal adhesions, lamellipodia, membrane ruffles and pseudopodia [13, 15, 16]. Copy number gain or amplification of the genomic locus of LASP1 at 17q12 was observed in 20-30% of human breast cancers [16] and identified as a hallmark of high-risk medulloblastoma [17]. The exact functions of LASP1 are still not clear, yet the protein overexpression was reported in approximately 50% of metastatic human colorectal cancer [18] and 63.7% of pancreatic cancer [19]. Silencing of LASP1 by RNA interference resulted in a significant inhibition of cell migration and proliferation [18–20] and attenuation of TGF-β mediated epithelial-mesenchymal transition (EMT)[18]. More recently, it has been shown that up-regulation of LASP1 created aggressive phenotypes of ESCC, thereby promoting cancer growth and metastasis [20].

In this study, we found that expression of PVT1 was significantly up-regulated in ESCC cancer tissues and cell lines and predicts poor prognosis of ESCC patients. Knockdown of PVT1 could inhibit ESCC cancer cell proliferation and migration *in vitro* as well as tumorigenesis *in vivo*. Moreover, we found PVT1 could act as a molecular sponge of miR-203 and LASP1. Our study provides the first evidence of the newly identified PVT1/miR-203/LASP1 axis in carcinogenesis and metastasis of ESCC and may serve as a candidate target for cancer therapy.

## RESULTS

### PVT1 expression is up-regulated in ESCC cancer tissues

**Figure 1:**
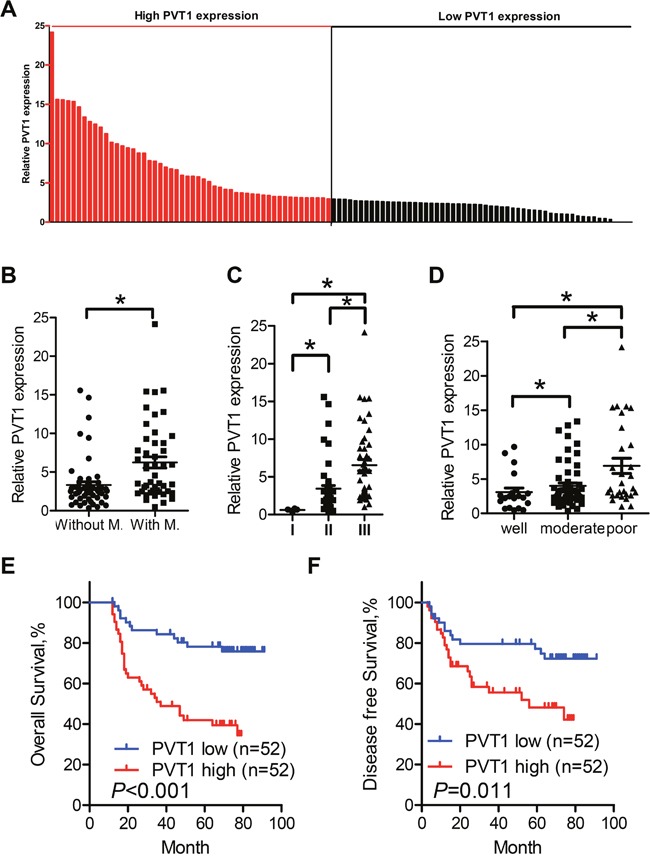
Relative PVT1 expression in cell lines and tissues assessed by qPCR and its clinical significance **A**. Overexpression (tumor/normal>2.0) of PVT1 was detected in 78.8% of ESCC tissues. Relative expression of PVT1 was detected in 104 pairs of clinical samples and normalized to GADPH. Data was presented as fold-change in tumor tissues relative to normal tissues. The red column was defined as overexpression. **B**. Relative expression level of PVT1 in ESCC cancer tissues with (n = 47) and without lymph node metastasis (n=57). PVT1 expression was lower in tissues without lymph node metastasis compared with tissues with lymph node metastasis. **C**. Relative expression level of PVT1 in different clinical stage. **D**. Relative expression level of PVT1 in samples with different differentiation state. The statistical differences between the two groups were analyzed using unpaired Student's t-test. **E**. Kaplan–Meier curves indicate 104 patients with high-level PVT1 expression (n=52) reported reduced overall survival time compared with patients with low-level PVT1 expression (n=52) (*P*<0.001, log-rank test) **F**. Kaplan–Meier curves indicate patients with high-level PVT1 expression reported reduced disease free survival time compared with patients with low-level PVT1 expression (*P*<0.001, log-rank test). Error bars indicate means ± S.E.M. *P < 0.05.

To investigate the role of PVT1 in ESCC progression, we detected the PVT1 expression levels in 104 paired ESCC cancer tissues and corresponding non-tumor tissues using qPCR, which was normalized to GAPDH. The transcript levels of PVT1 were significantly up-regulated (cancer/normal > 2.0) in 78.8% (82 of 104) of ESCC cancer tissues (Figure 1A), with the mean fold-change of 2.92 in tumor tissues compared with that in their normal counterparts. Higher PVT1 expression level was observed in tissues with lymph node metastasis than tissues without lymph node metastasis (Figure 1B). Also, the PVT1 expression level was markedly correlated with the TNM stage of ESCC cancer patients (Figure 1C) and tumor differentiation state (Figure 1D). These data indicated that dysregulated PVT1 expression might be related to ESCC pathogenesis.

### PVT1 creates aggressive tumor phenotypes and predicts adverse prognosis in ESCC patients

Next, we examined the correlation of PVT1 expression level with clinical pathological features of ESCC patients. The median level of PVT1 expression was used as a cutoff value to classify all 104 patients into two groups. The relationship between PVT1 expression and clinical pathologic factors were compared and summarized in Table 1. High PVT1 expression was significantly associated with poor differentiation status (*P* = 0.024) and TNM stage (*P* = 0.001). However, the relative PVT1 expression was not associated with other parameters such as age (*P* = 0.325), gender (*P* = 0.816), alcohol (*P* = 0.842), tobacco consumption (*P* = 0.837), tumor size (*P* = 1.000) nor N stage (*P* = 0.597). Kaplan-Meier analysis (Supplementary Table1) demonstrated that five year overall survival for patients with high PVT1 expression is 46 months, while is 76 months for those with low PVT1 expression (Figure 1D, Log-rank *P* < 0.001). Moreover, disease-free survival for patients with high PVT1 expression was significantly shorter than those with low PVT1 expression (Figure 1E, Log-rank *P* = 0.011). By univariate analysis, differentiation status (*P* = 0.007), N stage (*P* = 0.001), TNM stage (*P* = 0.002) and PVT1 expression level (*P* < 0.001) were identified as prognostic factors, while other clinical parameters were not significant prognosis factors (Table 2). Further analysis in a multivariate Cox proportional hazards model showed that only PVT1 expression was significantly correlated with overall survival in our study cohort (*P* = 0.005).

**Table 1:** The correlation between clinicopathological parameters and PVT1 expression

	PVT1 expression		*P*
	Low, n(%)	High, n(%)	
Age			
<60	31 (59.6)	25(48.1)	0.325
≥60	21(40.4)	27(51.9)	
Gender			
Male	13(25.0)	11(21.2)	0.816
Female	39(75.0)	41(78.8)	
Alcohol consumption			
Ever and current	30(57.7)	32(42.3)	0.842
Never	22(42.3)	20(58.7)	
Smoking status			
Ever and current	17(32.7)	19(36.7)	0.837
Never	35(67.3)	33(63.5)	
Tumor size			
<4cm	43(82.7)	44(67.3)	1.000
≥4cm	9(17.3)	8(32.7)	
Differentiation status			
Well or Moderate	44(84.6)	33(63.5)	0.024*
Poor	8(15.4)	19(36.5)	
N stage			
N0-N1	45(86.5)	42(80.8)	0.597
N2-N3	7(13.5)	10(19.2)	
TNM stage			0.001*
I-II	39(75.0)	21(40.4)	
III	13(25.0)	31(59.6)	

*P < 0.05.

**Table 2:** Univariate and multivariate analyses of various potential prognostic factors in ESCC patients

	Univariate analysis		Multivariate analysis	
	HR (95% CI)	*P*	HR (95% CI)	*P*
Age (<60/≥60)	1.24(0.68-2.26)	0.476	-	-
Gender (male/female)	0.85(0.43-1.69)	0.647	-	-
Tumor size (≥5cm/<5cm)	1.61(0.74-3.47)	0.228	-	-
Alcohol (Ever/never)	0.89(0.48-1.66)	0.716	-	-
Smoke (Ever/never)	0.90(0.49-1.68)	0.752	-	-
Differentiation (well, moderate/poor)	2.37(1.27-4.41)	0.007*	1.82(0.96-3.44)	0.065
N stage (N0-N1/N2-N3)	3.15(1.60-6.20)	0.001*	2.03(0.91-4.51)	0.084
TNM Stage(I-II/III)	2.67(1.45-4.91)	0.002*	1.39(0.66-2.91)	0.385
PVT1 (high/low)	3.65(1.87-7.14)	0.000*	2.75(1.35-5.59)	0.005*

HR: hazard ratio; CI: confidence interval; *P < 0.05.

### PVT1 promotes cell proliferation and migration *in vitro*

**Figure 2:**
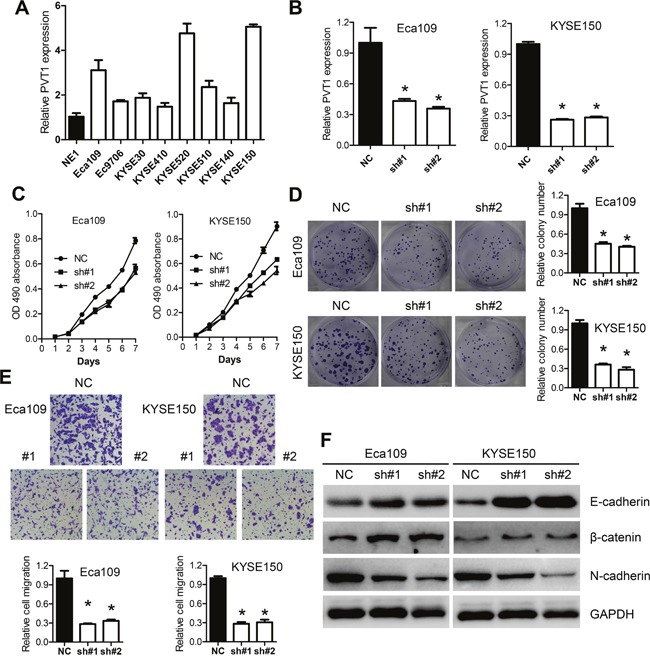
Knockdown of PVT1 inhibits ESCC cancer cells growth *in vitro* **A**. Expression level of PVT1 in ESCC cell lines (EC109, EC9706, KYSE30, KYSE410, KYSE520, KYSE510, KYSE140 and KYSE150) compared with that of the immortalized esophageal epithelial cell line NE1, data was presented as expression fold-change relative to NE1. **B**. Expression level of PVT1 in Eca109 and KYSE150 cells following transfection with NC, sh#1 and sh#2. sh#1 and sh#2 indicate the two shRNAs used in this study. **C**. Knockdown of PVT1 suppressed ESCC growth *in vitro*. MTS assays were performed to determine the proliferation of indicated cells. **D**. Colony-forming growth assays were also performed to determine the proliferation of EC109 and KYSE150 cells. The colonies were counted and captured. **E**. Representative pictures and quantification of migration assays in the indicated cells. **F**. Knockdown of PVT1 increased the level of epithelial markers such as E-cadherin, β-catenin while reduced the level of mesenchymal marker N-cadherin in Eca109 and KYSE150 cells. Error bars indicate means ± S.E.M. *P < 0.05.

We then evaluated the expression of PVT1 in a panel of ESCC cell lines. As shown in Figure 2A, overexpression of PVT1 was detected in ESCC cancer cell lines compared with that in the immortalized esophageal epithelium cell lines (NE1). Two effective interference target sequences, which was previously reported to be effective was used to construct lentivirus to silence the expression of PVT1 in Eca109 and KYSE150 cells [21]. A PVT1 expression vector was transfected into Eca9706 and KYSE140 cells. qPCR assays revealed that PVT1 expression was significantly reduced in Eca109 and KYSE150 cells (Figure 2B) and elevated in Eca9706 and KYSE140 cells (Supplementary Figure 1A). Next, MTS assays showed that knockdown of PVT1 expression significantly inhibited cell proliferation in both Eca109 and KYSE150 cell lines compared with the control cells (Figure 2C). Colony formation ability was also reduced by the silence of PVT1 expression in Eca109 and KYSE150 cells (Figure 2D). Consistently, overexpression of PVT1 significantly promoted proliferation and colony formation of Eca9706 and KYSE140 cells (Supplementary Figure 1B and Supplementary Figure 1C). Furthermore, the percentage of cells migrated to the lower chamber was significantly decreased in cells transfected with short hairpin RNA (sh#1 and sh#2) compared with that in the control cells (Figure 2E). Interestingly, western blot analysis indicated that knockdown of PVT1 resulted in an elevated level of the epithelial markers E-cadherin and β-catenin and reduced level of the mesenchymal marker N-Cadherin (Figure 2F). Altogether, our data demonstrated that knockdown of PVT1 could inhibit ESCC proliferation and migration *in vitro*.

### Knockdown of PVT1 suppresses ESCC tumor growth *in vivo*

**Figure 3:**
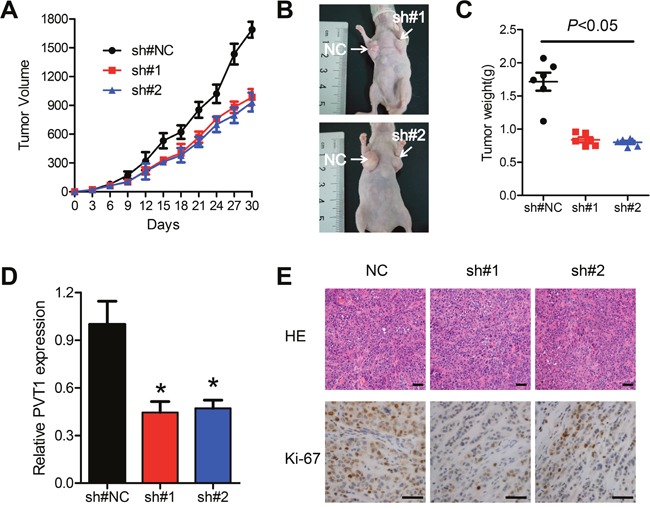
Knockdown of PVT1 suppresses ESCC tumor growth *in vivo* **A and B**. Lentivirus expressing the NC, sh#1 or sh#2 sequences were transfected into Eca109 cells, which were injected in the nude mice. Tumor volumes were calculated every other day after 4 days of injection. Bars indicate S.E.M. **C**. Tumor weights are represented as means of tumor weights ±S.E.M. **D**. qPCR was performed to determine the average expression of PVT1. **E**. Histopathology of xenograft tumors. The tumor sections were under H&E staining and immunohistochemistry staining using antibodies against Ki-67. Scale bars: 50μm. Error bars indicate means ± S.E.M. *P < 0.05.

To further determine whether the effects of PVT1 on ESCC, Eca109 cells transfected with either scramble (NC) or shPVT1 (#1, #2) into the axillary fossa of the nude mice and tumor size was measured every three days. In consistent with the *in vitro* results, tumor growth in shPVT1 groups was significantly slower than that in the scrambled group (Figure 3A and 3B). After 30 days, mice were killed, and the tumors were dissected out. Tumor weight in the shPVT1 group was significantly lighter than that in the scrambled group (Figure 3C). Moreover, the qPCR analysis demonstrated that the average level of PVT1 in shPVT1 group was lower than that in the scrambled group (Figure 3D). Immunohistochemical analysis indicated that the tumors dissected from the scramble groups showed a stronger Ki-67 expression than that in tumors from shPVT1 (Figure 3E). Altogether, our data further supported that knockdown of PVT1 suppressed ESCC tumor growth *in vivo*.

### PVT1 correlates negatively with expression of miR-203

**Figure 4:**
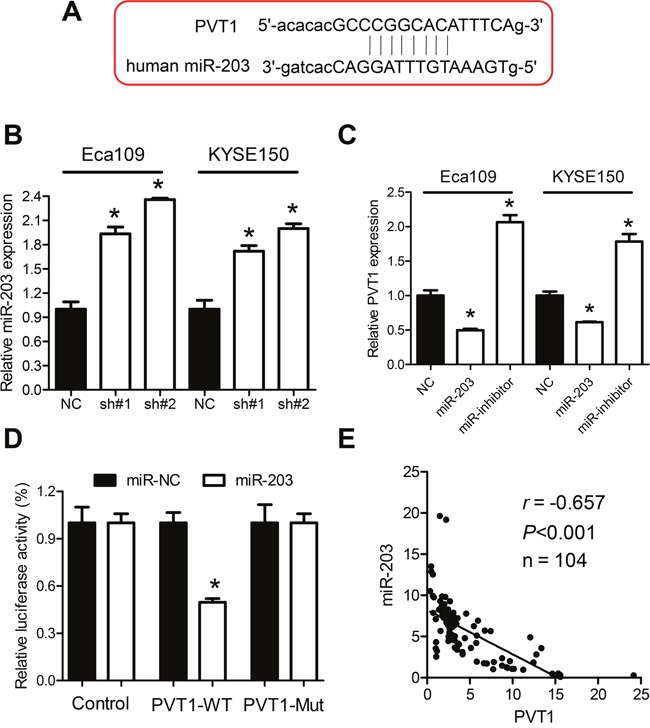
Regulation relationship between PVT1 and miR-203 **A**. Schematic representation of the predicted target sites for miR-203 in PVT1. **B**. Knockdown of PVT1 increased miR-203 expression in Eca109 and KYSE150 cells. **C**. Expression of PVT1 after upregulation or downregulation of miR-203 in Eca109 and KYSE150 cells. **D**. Luciferase reporter assay in HEK293T cells, co-transfected with the reporter plasmid (or the corresponding mutant reporter) and the indicated miRNAs. MiR-203 significantly decreased the luciferase activity in PVT1-wt but not in PVT1-mt. **E**. The expression of PVT1 was inversely correlated with the expression level of miR-203 in ESCC cancer tissues. Error bars indicate means ± S.E.M. *P < 0.05.

PVT1 has been previously reported to function as competitive endogenous RNA (ceRNA) or naturally occurring miRNA sponge to regulate gene expression. We first used the bioinformatic tools to search for the potential miRNA targets of PVT1. Interestingly, miR-203, which has been reported to be down-regulated in ESCC, could bind to PVT1 as shown in Figure 4A. Furthermore, the qPCR analysis indicated up-regulated expression of miR-203 after knockdown of PVT1 in Eca109 and KYSE150 cells (Figure 4B). Moreover, expression of PVT1 was significantly up-regulated after transfection with miR-203 inhibitor and down-regulated after transfection with the miR-203 mimics (Figure 4C). To examine the direct interaction of PVT1 and miR-203 experimentally, the predicted miR-203 binding site (PVT1-wt) and its mutant type (PVT1-mut) was subcloned downstream of the firefly luciferase gene and was designated as PVT1-wt and PVT1-mut, respectively. HEK293T cells were co-transfected with miR-203 mimics and PVT1-wt or PVT1-mut. MiR-203 produced a 51.4% decrease in relative luciferase activity compared with control vector-transfected cells in the PVT1-wt group (Figure 4D). However, there was no significant decrease in relative luciferase activity for cells transfected with miR-203 mimics compared with control vector-transfected cells in the PVT1-mut group (Figure 4D). To investigate whether there was an inverse correlation between PVT1 and miR-203 in ESCC cancer tissues, qPCR in 104 ESCC cancer tissues indicated a significant inverse correlation between PVT1 and miR-203 (Figure 4E, r = −0.657, *P* < 0.001). Altogether, these data indicated that PVT1 correlated inversely with expression of miR-203.

### PVT1 regulates miR-203 to modulate LASP1 in ESCC cancer cells

**Figure 5:**
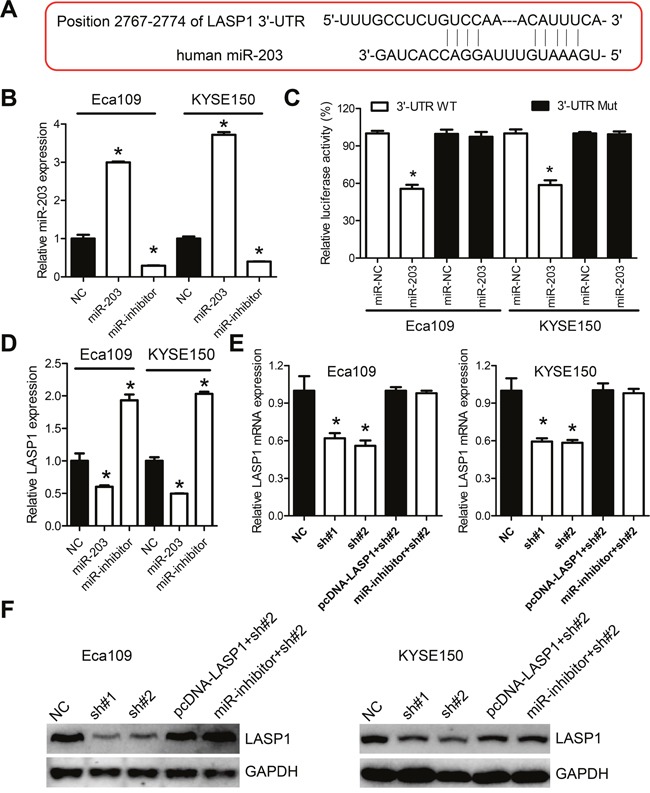
PVT1 regulates LASP1 expression by acting as molecular sponge for miR-203 **A**. Schematic representation of the predicted target site of miR-203 in LASP1 3'-UTR. **B**. qPCR analysis of miR-203 expression after transfection with the miR-203 mimic and miR-101 inhibitor in Eca109 and KYSE150 cells. **C**. Luciferase reporter assay for LASP1 mRNA 3'-UTR following miR-203 ectopic expression. **D**. Relative LASP1 mRNA level after transfected with control (NC), miR-203 mimic or inhibitor. **E**. Relative LASP1 mRNA level after knockdown of PVT1, or transfected with miR-203 inhibitor, or with the pcDNA-LASP1 lacking the 3'-UTR in Eca109 and KYSE150 cells. **F**. Western blot of LASP1 protein level after knockdown of PVT1, or transfected with miR-203 inhibitor, or with the pcDNA-LASP1 lacking the 3'-UTR in Eca109 and KYSE150 cells. Error bars indicate means ± S.E.M. *P < 0.05.

**Figure 6:**
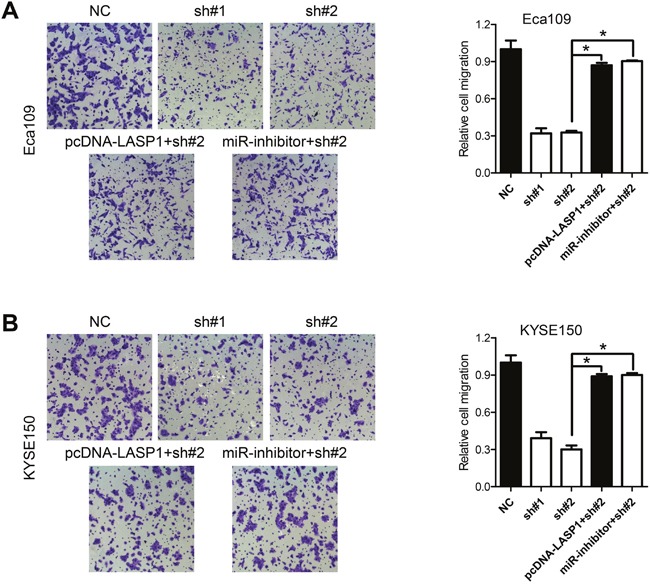
LASP1 expression mediated the biological effects exerted by PVT1 **A, B**. Cell migration assays of Eca109 and KYSE150 cells after knockdown of PVT1 and transfected with miR-203 inhibitor, or with the pcDNA-LASP1 lacking the 3'-UTR. Error bars indicate means ± S.E.M. *P < 0.05.

It has been well established that microRNAs function through regulation of downstream genes via binding to the 3'-untranslated region (3'-UTR) [22]. As we have demonstrated that PVT1 affect the expression of the miR-203 and miR-203/LASP1 axis has been reported in various human cancers [22, 23], it was thus reasonable to hypothesize that expression of LASP1 may be affected by dysregulated PVT1. Therefore, we firstly confirmed direct binding of miR-203 to the 3'-UTR of LASP1 (Figure 5A). qPCR analysis confirmed increased and reduced expression of miR-203 after transfection with miR-203 mimics or inhibitors, respectively (Figure 5B). Mimics of miR-203 markedly reduce the relative luciferase activity of LASP1 3'-UTR-wt, but not that of the LASP1 3'-UTR-wt in Eca109 and KYSE150 cells (Figure 5C). The mRNA level of LASP1 was decreased after transfection with miR-203 mimic, while increased after transfection with miR-203 inhibitor in Eca109 and KYSE150 cells (Figure 5D). Moreover, the decreased mRNA levels of LASP1 induced by PVT1 knockdown were significantly reversed by ectopic transfection of miR-203 inhibitor or a vector containing the coding sequences but lacking the 3'-UTR of LASP1 (pcDNA-LASP1, Figure 5E). Western blot analysis further demonstrated that LASP1 was regulated by PVT1/miR-203 axis (Figure 5F). Immunohistochemistry analysis showed that LASP1 was overexpressed in ESCC samples with high PVT1 expression (Supplementary Figure 2). Additionally, ectopic expression of miR-203 inhibitor or pcDNA-LASP1 attenuated the migration-suppression role of PVT1 in Eca109 and KYSE150 cells (Figure 6A and 6B). Taken together, PVT1 can modulate the expression of LASP1 via miR-203 in ESCC cells.

## DISCUSSION

Roles for lncRNAs as either tumor suppressor or oncogene have been tested in various cancer types [10–12]. Recent studies have found that lncRNAs may function as guides, decoys, scaffolds and tethers for other biological molecular [9] and are closely associated with malignant transformation of various cancers [24–26]. However, biological roles of PVT1 and the underlying mechanisms in ESCC remain unreported. In our study, we found that expression of PVT1 was significantly up-regulated in the ESCC cancer tissues than that in the normal counterparts and exerted oncogenic roles in ESCC. Wang F et al. found that PVT1 regulates stem cell property in hepatocellular caicinoma [26], while others reported that overexpression of PVT1 promotes tumor cell proliferation and migration [8] and cells with up-regulated PVT1 are resistance to cisplatin treatment [27]. PVT1 also mediates the oncogenic activity of Myc in colorectal cancer [6] and breast cancer [11]. These results demonstrated that PVT1 is an oncogenic lncRNA playing multifaceted roles in human cancers.

Mounting evidences have reported the ceRNA function of lncRNAs, whereby they act as a molecular sponge to inhibit miRNA expression [9, 24, 25, 28–30]. We therefore speculated that PVT1 may regulate ESCC cancer progression through silencing of specific miRNA and found that miR-203 was up-regulated after knockdown of PVT1. Expression of miR-203 in ESCC tumor tissues inversely correlated with that of PVT1. Also, a luciferase activity assay confirmed direct binding of PVT1 with miR-203. On the other hand, miR-152 [29] and miR-200b [30] have been reported to be regulated by PVT1 in hepatic stellate cells and cervical cancer cells, respectively. These data implied that interaction of PVT1 with miRNAs was cell and tumor type specific.

Down-regulated miR-203 has been reported in breast cancer [22] and head and neck squamous cell carcinoma (HNSCC) [31]. In ESCC, expression of miR-203 in cancer tissues is remarkably lower than that in nontumorous tissues and miR-203 suppressed migration and invasion of ESCC cells via downregulation of LASP1 [23]. This prompted us to explore whether overexpression of PVT1 was associated with dysregulation of LASP1 in ESCC. We first found that shRNA-mediated silence of PVT1 resulted in down-regulation of LASP1, which could be reversed by transfection of miR-203 inhibitor or ectopic expression of LASP1 lacking the 3'-UTR. Furthermore, the inhibitor of miR-203 attenuated the migration-suppression ability of PVT1 knockdown in ESCC cells. These data revealed that PVT1 exerts oncogenic roles in ESCC at least in part by LASP1.

In conclusion, we explored expression and biological effects of PVT1 as well as the underlying mechanisms in ESCC in this study. Our results indicated that PVT1 was up-regulated in ESCC and predicts overall prognosis. Knockdown of PVT1 inhibited proliferation and migration of ESCC *in vitro* and suppressed tumor growth *in vivo*. Meanwhile, PVT1 acted as the molecular sponge to regulate expression of miR-203 and LASP1. Our data provided the first evidence that PVT1 promoted ESCC progression via activation of the miR-203/LASP1 axis.

## MATERIALS AND METHODS

### Human tissue specimens and cell culture

Fresh-frozen ESCC cancer tissues and paired normal esophageal epithelial tissues were obtained from 104 patients who were undergoing surgery at the Cancer Center of Guangzhou Medical University from 2007 to 2009. Corresponding normal esophageal epithelial tissues were taken from tissues that were located 5cm away from tumor margin. All the samples were immediately snap-frozen in liquid nitrogen and stored at –80°C until RNA extraction. All the patients did not receive any treatment before the operation. The study was approved by the ethics committee of the Cancer Center of Guangzhou Medical University and informed consent was obtained from all patients. Each patient was followed up regularly every three months after surgery. The clinic pathological characteristics including age, gender, tumor size, differentiation, lymph node invasion and TNM stage were recorded. Overall survival was defined as the time from the date of surgery to the date of death or last contact.

Human embryonic kidney (HEK) 293T cells, human ESCC cell lines KYSE30, KYSE410, KYSE520, KYSE510, KYSE140, and KYSE150, were purchased from the Deutsche Sammlung von Mikroorganismen und Zellkulturen (DSMZ, Braunschweig, Germany). The ESCC cell lines Eca109, Eca9706 and esophageal epithelial cell NE1 was a kind gift from Dc. Guan XY from Sun Yat-sen University Cancer Center. The ESCC cell lines were grown in Dulbecco's modified Eagle medium (Invitrogen, Carlsbad, California, USA) supplemented with 10% fetal bovine serum (HyClone, Logan, Utah, USA) at 37°C with 5% CO2. NE1 and NE3 cells were maintained in a 1:1 mixture of defined keratinocyte serum-free medium with growth supplements and EpiLife medium with 60μM Calcium (Invitrogen, Carlsbad, California, USA).

### RNA extraction and qPCR analysis

Total RNA was extracted from tissue samples and cells using Trizol reagent (Life Technologies, Carlsbad, CA) according to the manufacturer's protocol. RNA was reverse transcribed to cDNA by using a Reverse Transcription Kit (Takara, Dalian, China). Real-time PCR was performed with SYBR Green (Takara, Dalian China). GAPDH was used as the internal control for mRNA or lncRNAs. Each sample was analyzed in triplicate. The following primers were used for the quantitative PCR:

PVT1 forward: 5'-GCCCCTTCTATGGGAATCACTA-3'

 reverse: 5'-GGGGCAGAGATGAAATCGTAAT-3'

LASP1 forward: 5'-TGCGGCAAGATCGTGTATCC-3'

 reverse: 5'-GCAGTAGGGCTTCTTCTCGTAG-3'

GAPDH forward: 5'-GGAGCGAGATCCCTCCAAAAT-3'

 reverse: 5'-GGCTGTTGTCATACTTCTCATGG-3'

To detect miR-203 expressions, the qPCR reactions were performed using the TaqMan MicroRNA Assays (Applied Biosystems, Foster City, CA) according to the manufacturer's instructions. U6 snRNA was used to normalize the relative abundance of miRNAs. Fold changes of PVT1, LASP1 and miR-203 were determined using the relative quantification 2^-ΔΔCT^ method.

### Stable cell lines construction

Eca109 and KYSE150 cells were seeded in the 6-well plate before they were transfected with lentivirus expressing shRNA or scramble sequence. After 48h, cells were selected with medium with 3μg/ml puromycin for another 72h. Then the cells survived and with GFP expression were picked as NC, sh#1, and sh#2 and used for subsequent assays.

### Cell transfections

Vectors expressing the coding sequence of LASP1 without the 3'-UTR, miR-203 mimics, and miR-203 inhibitor were obtained from GenePharma. Eca109 and KYSE150 cells were transfected with miR-203 mimics or miR-203 inhibitor using Lipofectamine RNAiMAX at a final concentration of 10 nM according to the manufacturer's protocol. After 48h, cells were trypsinized for qPCR assay or proteins were extracted for western blot analysis.

### Cell proliferation and migration assays

Cell proliferation was tested with the MTS kit (Promega) according to the manufacturer's instruction. The absorbance was measured at the wavelength of 490nm on a Synergy™ Multi-Mode Microplate Reader (Biotek, Vermont, USA). For colony formation assay, a certain number of transfected cells were placed in each well of a six-well plate and maintained in proper media containing 10% FBS for two weeks, during which the medium was replaced every four days. Colonies were then fixed with methanol and stained with 0.1% crystal violet (Sigma) for 10 minutes. Colony formation was determined by counting the number of stained colonies. The cell migratory potential was evaluated using transwell assays according to a previous report [10].

### Animal work

Four-week athymic female BALB/c mice were housed and maintained in laminar airflow chambers under specific pathogen-free conditions. The Eca109 NC, sh#1 and sh#2 cells were suspended in PBS at a final concentration of 1 × 10^7^ cells/ml separately, a volume of 100μl of suspended cells was subcutaneously injected into the axillary fossa of each mouse. At 30 days post-injection, the mice were sacrificed by cervical dislocation in diethyl ether anesthesia, and the tumors were dissected out and weighed. The primary tumors were excised, and tumor tissues were used to perform qPCR analysis of PVT1 expression levels and immunostaining analysis of Ki-67 protein expression. All the animal experiments were performed according to the National Institutes of Health animal use guidelines on the use of experimental animals.

### Reporter vector construction and luciferase reporter assay

The bioinformatics database (Starbase v2.0) was used to search for potential microRNAs that can bind to PVT1. Oligonucleotides containing target sequences of PVT1 were amplified and cloned into pmirGLO plasmids (pGL3-PVT1-wt). To obtain the mutated constructs, the fragments harboring the mutated target region were synthesized by GenePharma (pGL3-PVT1-mut). Empty plasmid pmirGLO was regarded as a negative control. Luciferase reporter plasmids plus miR-203 mimic or miR-NC were co-transfected into HEK293T cells using Lipofectamine RNAiMAX. Forty-eight hours after transfection, relative luciferase activity was examined in a luminometer using a Dual-Luciferase Reporter Assay System (Promega, Madison, WI, USA). To confirm the direct regulating relationship between miR-203 and LASP1, the full-length 3'-UTR of the LASP1 mRNA (3'-UTR-wt) and a mutant variant (3'-UTR-mut) were amplified by PCR and cloned into the XbaI site of a pGL3-basic vector (Promega). Cells were cultured in a six-well plate and then transfected with the miR-203 expression vector or the negative control (NC) (750ng/well), the pGL3 reporter vector (250ng/well) and the pRL-TK luciferase reporters (25ng/well) using Lipofectamine 3000 (Invitrogen). Luciferase activity levels were measured using the Dual-Luciferase Reporter Assay Kit following the manufacturer's instructions.

### Western blot analyses and immunohistochemistry assays

Total proteins were extracted from tissues or cells and separated using SDS-PAGE gels. Antibodies for LASP1, E-cadherin, β-catenin and N-cadherin were purchased from Cell Signaling Technology, and an anti-GAPDH antibody (1:2000; Santa Cruz Biotechnology, USA) was used as a loading control. The anti-LASP1 antibody used for immunohistochemistry assays was purchased from Abcam (Cambridge, Massachusetts, USA). The procedure of Western blot and immunohistochemistry analysis was performed as previously described [24].

### Statistical analyses

Each biological experiment was performed in triplicate and repeated three times. Data are shown as mean ± standard error of the mean (S.E.M). Statistical significance was tested using the SPSS software package (version 16.0, SPSS Inc.) or GraphPad Prism 5.0 by Student's t-test (two-tailed), Chi-square test and One-way ANOVA analysis as appropriate. Survival curves were generated using the Kaplan-Meier method and assessed using the log-rank test. The Cox proportional hazard regression model was performed to identify independent prognostic factors. *P* < 0.05 was considered as statistically significant.

## SUPPLEMENTARY MATERIALS FIGURES AND TABLES


