# Supplementary material for: Upregulation of the long non-coding RNA PVT1 promotes esophageal squamous cell carcinoma progression by acting as a molecular sponge of miR-203 and LASP1
Source: Oncotarget. 2017 Mar 3;8(21):34164–76. doi: 10.18632/oncotarget.15878 (PMC5470958; doi:10.18632/oncotarget.15878)
Supplement: Supplementary file 1 [file oncotarget-08-34164-s001.pdf]

Upregulation of the long non-coding RNA PVT1 promotes esophageal squamous cell carcinoma progression by acting as a molecular sponge of miR-203 and LASP1

SUPPLEMENTARY FIGURES AND TABLE

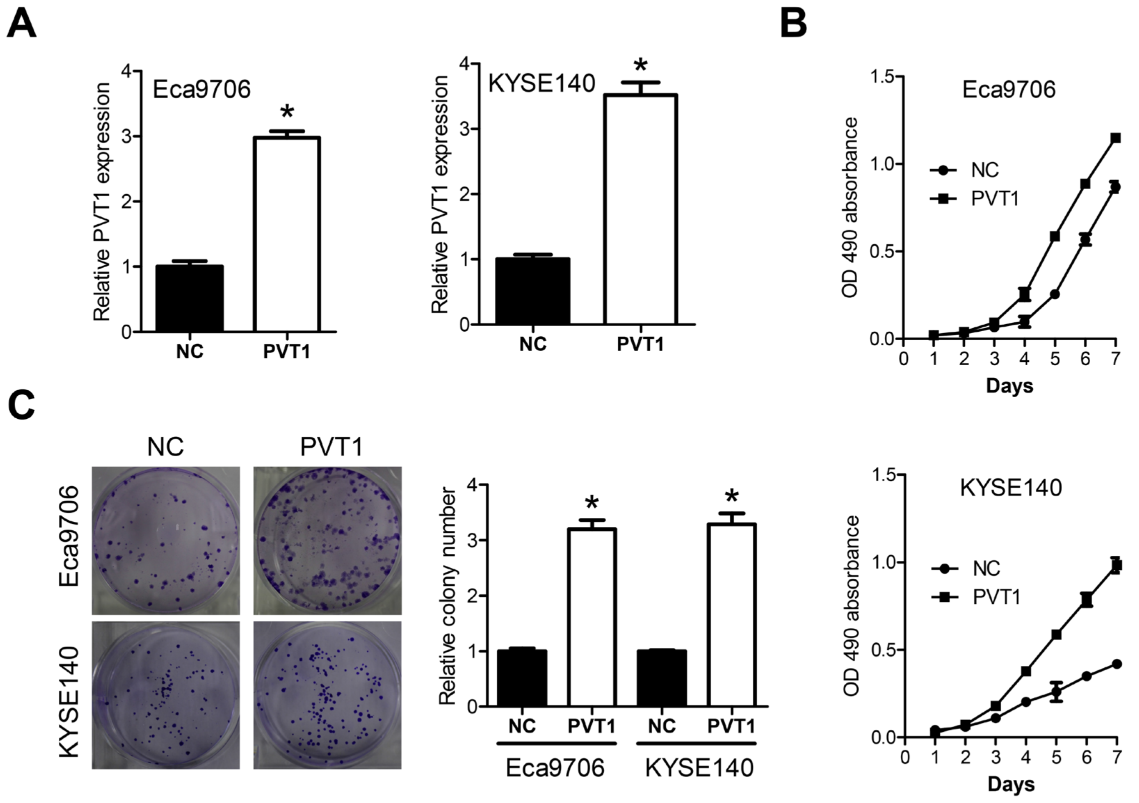

Supplementary Figure 1: Overexpression of PVT1 promotes proliferation of ESCC cells. A. Expression level of PVT1 in Eca9706 and KYSE140 cells following transfection with NC and PVT1 vector. **B.** Overexpression of PVT1 promoted ESCC growth in vitro. MTS assays were performed to determine the proliferation of indicated cells. **C.** Colony-forming growth assays were also performed to determine the proliferation of Eca9706 and KYSE140 cells . The colonies were counted and captured. Error bars indicate means ± S.E.M. *$P < 0.05$.

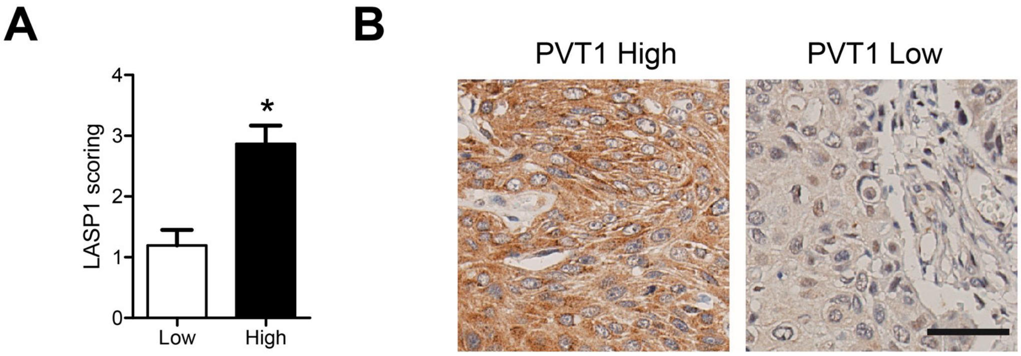

Supplementary Figure 2: Protein expression of LASP1 was elevated in samples with high PVT1 expression. **A.** Scoring of LASP1 immunostaining in ESCC samples with high or low PVT1 expression. **B.** Representative immunostaining of LASP1 in samples with high or low PVT1 expression. Scar bars: 50μm. Error bars indicate means ± S.E.M. $^{*}P < 0.05$.

Supplementary Table 1: Actual patient number involved in survival analysis

	OS	DFS
PVT1 high	52	52
PVT1 low	52	52
